# Supplementary material for: Cold sensitivity of TRPA1 is unveiled by the prolyl hydroxylation blockade-induced sensitization to ROS
Source: Nat Commun. 2016 Sep 15;7:12840. doi: 10.1038/ncomms12840 (PMC5027619; doi:10.1038/ncomms12840)
Supplement: Supplementary Information — Supplementary Figures 1-6, Supplementary Table 1 [file ncomms12840-s1.pdf]

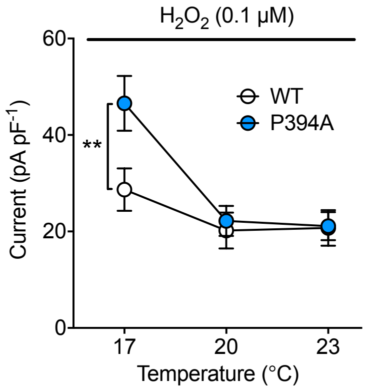

Supplementary Figure 1 | Cold stimulation induces hTRPA1-P394A activation in the presence of H_2O_2. Shown are temperature-current relationships at +80 mV obtained from whole-cell patch-clamp recordings as in Fig. 1. $n = 8$. $^{**}P < 0.01$; two-way ANOVA with Sidak's *post hoc* test. Data are expressed as mean ± s.e.m.

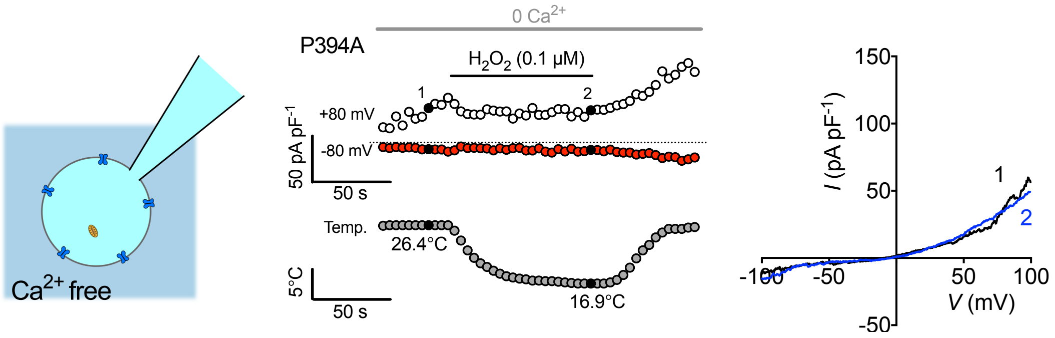

Supplementary Figure 2 | Cold stimulation does not induce hTRPA1-P394A activation in the absence of extracellular Ca^{2+} ions. A hTRPA1-P394A-expressing cell was exposed to cold and 0.1 μM H_2O_2 in a Ca^{2+}-free bath solution and analyzed by whole-cell patch clamp recordings. Shown are a schematic diagram of whole-cell patch clamp configurations used in this experiment, a whole-cell recording from a hTRPA1-P394A-expressing cell (a representative of six cells), and current-voltage relationships at the times indicated by the black-filled circles.

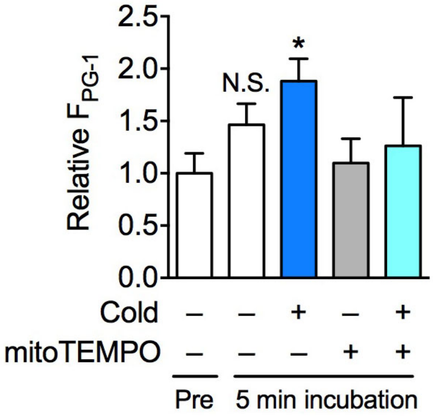

Supplementary Figure 3 | Cold stimulation induces H_2O_2 production in HEK293 cells. HEK293 cells were incubated at 37°C or on ice for 5 min. The cells were then collected, and the intracellular H_2O_2 level was measured using PG-1, a fluorescent probe with high selectivity for H_2O_2. MitoTEMPO was preloaded with PG-1. $n = 6$. N.S., not significant; $*P < 0.05$ versus pre-incubation sample (Pre); unpaired t test. Data are expressed as mean ± s.e.m.

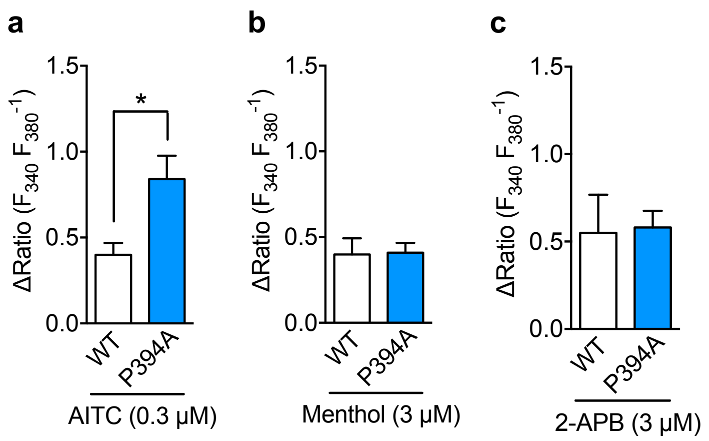

Supplementary Figure 4 | Relief from prolyl hydroxylation of hTRPA1 enhances the response to AITC, but not menthol and 2-APB. The hTRPA1-P394A-expressing cells were exposed to three TRPA1 agonists and analyzed using Ca^{2+} imaging experiments. Shown are statistical analyses of the $[Ca^{2+}]_i$ responses evoked by 0.3 μM AITC (**a**), 3 μM menthol (**b**), or 3 μM 2-APB (**c**) in hTRPA1-WT- or hTRPA1-P394A-expressing cells (*n* = 5–6). $^*P < 0.05$; unpaired *t* test. All data are expressed as mean ± s.e.m.

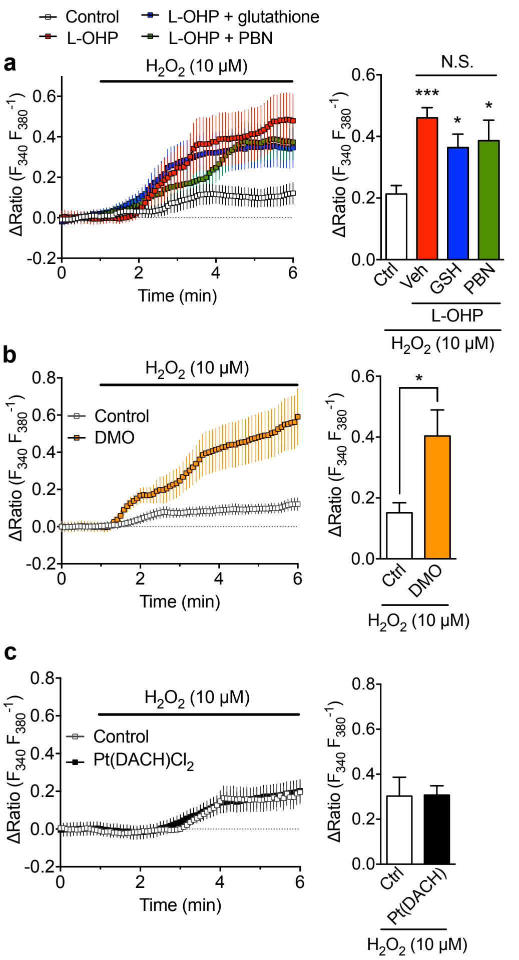

Supplementary Figure 5 | L-OHP and DMO, but not Pt(DACH)Cl_2, elicit hTRPA1 sensitization. Shown are representative traces of H_2O_2-evoked $[Ca^{2+}]_i$ responses in hTRPA1-WT-expressing cells pretreated with or without 100 μM L-OHP (**a,** *n* = 29–47 cells), 30 μM DMO (**b,** *n* = 44–57 cells), or 30 μM Pt(DACH)Cl_2 (**c,** *n* = 36–58 cells) for 2 h, and the statistical analysis (**a,** *n* = 5–12; **b,** *n* = 4; **c,** *n* = 6). Note that glutathione (1 mM) or PBN (10 mM) were co-pretreated for 2 h with L-OHP and washed out before starting Ca^{2+} imaging experiments. N.S., not significant; $*P < 0.05$, $***P < 0.001$ versus control (Ctrl), if not otherwise indicated; one-way ANOVA with Tukey's *post hoc* test (**a**) and unpaired *t* test (**b, c**). All data are expressed as mean ± s.e.m.

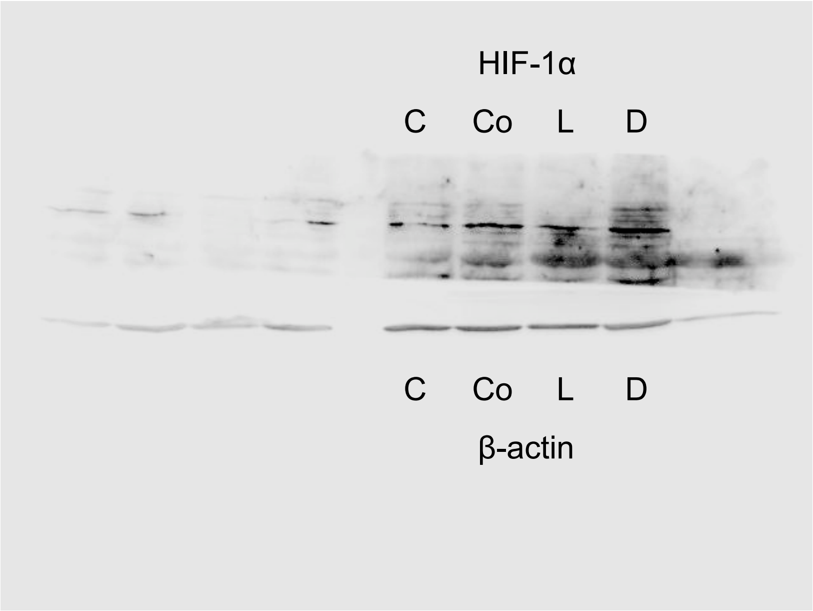

Supplementary Figure 6 | Full image of western blotting experiment. HEK293 cells western blotting result in Figure 6a. C, control; Co, $CoCl_2$; L, L-OHP; D, DMO.

Supplementary Table 1 | Primer sequences used for mTRPA1 cloning.

Primer name	Sequences (5'→3')	Restriction sites used for cloning
mTRPA1(+)28ReKoz	aaactcgaggatccaccatgaagcgcgg	XhoI
mTRPA1(-)1226	atctgcataaactcaggccgcaaatttcttagtc	
mTRPA1(+)1059	ctctccacttattttagcaacagcttctgcatcc	
mTRPA1(-)2509	tgaggaacaagggcaacacgaagatgatac	
mTRPA1(+)2247	caaaatacagcctggaatggccttcaattc	
mTRPA1(-)3405Re	aaacccggggtcgacctaaaagtccg	SalI

The number in the primer name represents the target locus of mTRPA1 sequence.
